# Depressive Syndromes in Autoimmune Disorders of the Nervous System: Prevalence, Etiology, and Influence

**DOI:** 10.3389/fpsyt.2018.00451

**Published:** 2018-09-25

**Authors:** Yanjun Liu, Xiangqi Tang

**Affiliations:** Department of Neurology, The Second Xiangya Hospital, Central South University, Changsha, China

**Keywords:** depression, anxiety, autoimmunity, nervous system disorder, etiology

## Abstract

Autoimmune diseases of the nervous system (ADNS) consist of a group of severely disabling disorders characterized by abnormal immune attack against protein components of the nervous system. This type of attack behavior may occur in the central or peripheral nervous system, and in the neuromuscular junction, resulting in neuronal damage, axonal injury, demyelination or destruction of the neuromuscular junction. While the neurological deficits of patients with ADNS have received significant research attention, the manifestation of depression tends to be ignored. In fact, depressive manifestation is common in ADNS and adds significant burden upon patients suffering from this disease. Here, we systematically reviewed the current literature to highlight the prevalence, etiology and influence of depressive manifestation in ADNS. Most autoimmune diseases of the nervous system are discussed in this paper, from multiple sclerosis, acute disseminated encephalomyelitis and autoimmune encephalitis to acute myelitis, neuromyelitis optica, Guillain-Barré syndrome and myasthenia gravis. Depressive symptoms usually develop as a comorbidity during the course of disease, but sometimes exist as a primary presentation of the disease. Psychosocial factors, long periods of disablement and chronic pain are the three most common causes of depressive symptoms in many chronic conditions, particularly in peripheral neuropathy. Furthermore, the higher prevalence of depressive symptoms in ADNS suggests that immunological dysregulation may contribute to the elevated morbidity of depression. Finally, structural lesions of the brain, and some medications for ADNS, are also thought to precipitate depressive states in ADNS.

## Introduction

Over the last few years, autoimmunity has been increasingly confirmed in a variety of neurological disorders involving both the central and peripheral nervous system. The concept of autoimmune disorders of the nervous system (ADNS) describes a broad spectrum of severely disabling disorders characterized by abnormal immune attack against protein components of the nervous system, which are mistaken as an invading antigen (1). Research has identified some of the novel autoantibodies involved, such as aquaporin 4 antibody (AQP4-Ab) and anti N-Methyl-D-Aspartate receptor antibody (NMDAR-Ab) (2). Such immune attack behavior may occur in the central or peripheral nervous system or the neuromuscular junction, resulting in neuronal damage, axonal injury, demyelination or destruction of the neuromuscular junction.

The complexity of autoimmune diseases, and the disabling effect of nervous system disorders in ADNS patients, requires timely and appropriate treatment, and careful clinical management. However, neurological deficit dominates the clinical profiling and outcome assessment of such patients. Depressive disorders or symptoms tend to be ignored, which may place a heavy burden on the patients affected (3). Indeed, various physical symptoms of the disease may be enhanced by a depressive condition, thus leading to poorer prognosis and a longer course of disease. Furthermore, overlapping neuropsychiatric presentations, such as fatigue and psychomotor retardation, could lead to an inadequate diagnosis and treatment of depression in affected patients. Depressive syndromes can also exert a strong negative effect upon a patient's quality of life and adherence to treatment. Conversely, severe physical symptoms can also reduce the level of depressive syndrome. Approximately 10–27% of patients with other types of neurological disorders also suffer from depression, including those with Parkinson's disease, Alzheimer's disease and post-stroke patients (4–6). However, these figures underestimate the noticeable likelihood of clinically significant depressive symptoms acting as a comorbid condition in some types of neurological disease, especially in patients with ADNS.

Depressed mood, lack of energy, pleasure loss, sleep disturbance and suicide ideation or attempts are the mainly depressive presentation. And mental retardation or agitation, loss of confidence are also frequently seen in ADNS. All these depressive symptoms can emerge with other psychiatric symptoms. The high incidence and negative influence of depressive syndromes in ADNS deserves increased clinical attention. Consequently, we systematically reviewed the current literature and aimed to highlight the prevalence and influence of depressive syndromes in ADNS. In psychology, different from primary depression, secondary depression describes the depressive state caused by other diseases including somatic disease-related depression and drug-induced depression. Therefore, we discuss the possible mechanisms underlying the elevated frequency of depression in ADNS.

Psychosocial factors and long duration are recognized as the basic causal factors of depression. Additionally, emerging neuroimmune inflammatory theories of depression support the doubtful causal connection between immunological dysregulation and comorbid depression in ADNS (7). Structural brain immune damage, as well as specific drugs for ADNS, is also thought to precipitate depressive states in patients with ADNS.

## Prevalence and profile

Most autoimmune diseases of the nervous system are discussed in this paper, including multiple sclerosis, acute disseminated encephalomyelitis, autoimmune encephalitis, acute myelitis, neuromyelitis optica, Guillain-Barré syndrome and myasthenia gravis. For the convenience of description, autoimmune diseases of the nervous system discussed in this paper are divided into four parts according to the affected area; the brain, spinal cord, peripheral nervous system and neuromuscular junction.

### Depressive syndromes in ADNS involving the brain

Multiple sclerosis, acute disseminated encephalomyelitis and autoimmune encephalitis are grouped together because of their involvement with brain tissue, among which lesions in specific areas may correlate with changes in mental condition and behavior.

Multiple sclerosis (MS) is an acquired demyelinating disorder of the central nervous system (CNS) caused by an autoimmune response, affecting one in 1,000 individuals in high-prevalence areas and making MS the most frequent entity of neurological disability in young people (8). The association between MS and depression has been acknowledged for some time (9). Patients with MS have a higher risk of developing depressive syndromes which can emerge at any stage of the disease course. Depressive syndromes in MS are characterized by low mood along with predominant fatigue and sleep disturbance in the depressed background, but with a lower frequency of comorbid anxiety disorder(10). Some MS patients report psychiatric symptoms, such as hallucination, suicidal ideation and compulsive-obsessive symptoms after medication for MS or depression(11). About 15% MS patients present with depressive symptoms before neurological MS symptoms and one in four to half of the patients show depressive state within 1 year after MS onset (9, 12). 75% MS patients have a delay in diagnosis as a consequence of depressive symptoms(13). However, few studies have reported the incidence of depressive syndrome in MS patients. Furthermore, previous results relating to the prevalence of depression and anxiety in MS show significant discrepancy, ranging from 14 to 54%, and 1.24 to 36%, respectively (14–16). This could be explained by the heterogeneity of methodological issues and potential selection bias, such as differences in definitions, the diagnostic criteria used, and the size and source of the population studied. Moreover, some previously-published studies focused on lifetime depression rather than current depression, although the distinction between these two conditions was not always explicit. This may partly contribute to the differential results described in the present literature. Besides the elevated prevalence, a considerable number of studies show an increasing depressive symptom score in MS, compared with other chronic diseases, though the score shows no correlation with the level of neurological impairment (17). Furthermore, Boeschoten et al. found no statistical difference in depression severity and clinical profiles when evaluating different symptoms between depressed patients with MS (*n* = 83) and without MS (*n* = 782) (18). This finding showed that the criteria for simply identifying depression are also suitable for evaluating depression in MS.

Over the last decade, significant light has been shed on cognitive and psychiatric manifestations in MS, however these aspects have largely ignored acute disseminated encephalomyelitis (ADEM), an inflammatory demyelinating disorder of the CNS affecting the subcortical white matter and, to a lesser degree, the gray matter (19). ADEM is the most common cause of immune-mediated encephalitis and typically develops a monophasic course with various grouped symptoms of fever, headache, meningitis, seizures, spasticity, and psychosis (20, 21). Psychiatric symptoms in patients with ADEM include depression, unspecific behavior changes and irritability. In addition, an increasing number of cases show that ADEM patients present with depression alone before the development of neurological symptoms (22–24).

Autoimmune encephalitis (AE) refers to a group of newly identified non-infectious encephalitis conditions which feature autoantibodies against neuronal cell-surface or synaptic proteins (25). Unlike other forms of infectious encephalitis, autoimmune encephalitis presents with pronounced psychotic and non-psychotic mood symptoms during the initial phase or during the course of the disease. Among the wide range of psychiatric symptoms presented, depressive syndromes are frequently seen in AE. These symptoms often show limited and transient effects in response to anti-depressive drugs and features can vary in different AE sub-groups. Anti-NMDAR encephalitis, arguably one of the best described subtype of AE, mainly manifests with depressive symptoms at onset, including depressed mood or depressed mood accompanied by anxiety, mood lability, apathy, sleep disorder and suicide attempts (26). Agitation and panic attack are another aspect of mood disorders in anti-NMDA receptor encephalitis and always accompanied by aggressive behavior. These emotional disturbances proceed to develop over the course of the disease. The depressive syndrome in AE with leucine-rich glioma inactivated 1-Ab (LGI1) or γ-amino-butyric acid B-receptor-Ab GABA(B)R-Ab can be psychotic. Depression can also develop with other psychiatric symptoms including visual or auditory hallucinations and obsessive disorders (27). In AE, the forebrain and limbic system and, in particular, the hippocampus, are severely affected (28, 29). The psychiatric presentation in AE suggests that the destruction of a specific protein, or a specific structure in the brain, may be correlated to depression episodes.

### Depressive syndromes in ADNS involving spinal cord

Transverse myelitis (TM) and neuromyelitis optica (NMO) represent different types of autoimmune inflammatory demyelinating disorders of the CNS, and are characterized by predominant involvement of the spinal cord with no cerebral or only optic nerve lesions (30). The depressive comorbid conditions in TM and NMO have received less attention than MS, although many studies have highlighted its relationship with quality of life (31, 32). In fact, depressive morbidity is very common in these disease, with 17% of the patients with TM suffering from depression (33). Furthermore, TM patients with psychological morbidity are more disabled than those who do not have such additional morbidity (33). In one paper, patients with NMO were found to exhibit a similar prevalence (point prevalence 16%; lifetime prevalence 46%), along with features of cognitive impairments and depression, as MS patients, indicating a significant psychiatric burden (34). Another striking result was that nearly half of the NMO patients in this previous study reported recurrent depression and suicidality, which may be partly attributed to the psychological impairment experienced by these patients (34, 35). Fatigue and neuropathic pain, as the other common complaints, show overlapping interplay with the development of depression and may exacerbate the scale of depression (36). This is exemplified by Chavarro's research in which the scale of depression was moderately correlated with neuropathic pain, although this relationship was confounded by different levels of fatigue(37).

### Depressive syndromes in ADNS involving the peripheral nervous system

Guillain-Barré syndrome (GBS) and chronic inflammatory demyelination polyradiculoneuropathy (CIDP) are both types of autoimmune-mediated peripheral neuropathy but develop on different clinical courses (38, 39). Previous research has demonstrated that the autoimmune response in GBS is triggered by molecular mimicry between microbial and nerve antigens and leads to demyelination and axonal damage (40). Strength and sensory deficits tend to depict the clinical profile when assessing patients with GBS and CIDP. A substantial number of patients with GBS or CIDP were observed to experience depressive episodes and increasing attention has been afforded to the burden of depressive syndromes and their influence on a patient's quality of life (41–43). In a population-based cohort study on the risk of psychiatric disorders in GBS, the hazard ratio (HR) of 4,548 GBS patients with regards to the development of psychosis was 4.320 (adjusted HR, 95% confidence interval (CI): 3.852–4.842, *p* < 0.001), and in depressive disorder was 4.834 (*p* < 0.001), in comparison to a control group (44). In other words, patients with GBS had a 4.8-fold elevated risk of developing depressive disorders. Data from the Dutch Society of Neuromuscular Disorders showed that nearly 6.7% of patients with GBS, and 9% of patients with GBS or CIDP, suffered depression (45). Furthermore, among the 49 most severely affected patients, anxiety (82%), depressive symptoms (67%) and brief reactive psychosis (25%) were observed (46). It is evident that among patients with GBS, the occurrence and severity of depressive syndromes are dependent on the severity of the neurological deficit. Depressive symptoms may be, to some extent, understood as a result of severe dysfunction in other words, the loss of movement and communication. In addition, depression, along with anxiety and fatigue, usually present as a residual symptom but not as the initial symptom, occurring during the recovery phase following the acute phase (47). This opinion was supported by another study which arrived at the conclusion that psychological distress and depressive symptoms were present at 3 months but improved significantly 3–6 months after disease onset (48). It is noteworthy that in a physical training study of patients with GBS and CIDP, simple physical exercises were shown to significantly relieve anxiety and depression (49).

### Depressive syndromes in ADNS involving the neuromuscular junction

Myasthenia gravis (MG) is an autoimmune disorder featuring specific autoantibodies which target the acetylcholine receptor on the post-synaptic membrane of the neuromuscular junction. This disease is characterized by a chronic and fluctuating process of muscle weakness and fatigue, causing multiple symptoms, such as eyelid ptosis, swallowing difficulties and limb weakness (50). The psychiatric symptoms of MG can be complicated and can coincide with other symptoms of MG, such as fatigue and shortness of breath, leading to inadequate recognition. Sometimes, these symptoms develop during the course of the disease, causing misdiagnosis and an unnecessary intensive drug treatment (51, 52). Although data are limited, affective disorder, particularly depressive disorder, appears to be the most frequent psychiatric manifestation in MG, with the frequency of depressive disorders ranging from 17 to 50% (53, 54). The large discrepancy in these figures could be attributed to heterogeneity in methodology and the different criteria adopted. More female patients have been observed to exhibit these presentations (57% in women vs. 35% in men) (55). In a Japanese cross-sectional study involving six neurological centers, it was reported that unchanged post-intervention status, dose of oral prednisolone, disease duration and MG composite were independent factors associated with depressive condition in MG (54). However, all of the assessments carried out in this study were performed by a neurologist without the assistance of a psychiatrist. Furthermore, it is uncertain whether the correlation of oral prednisolone dose was related to the side effects of prednisone, or to the higher MG severity associated with a higher prednisone dosage.

Depressive symptoms in ADNS are seldom reported in isolation and always emerge as a symptom cluster. Emotional disturbance frequently co-occur with fatigue, sleep disturbance and can be significantly strengthened by each other or somatic symptoms, such as pain. Depressive syndromes in ADNS involving brain tissues are more likely accompanied with other psychiatric symptoms including compulsive-obsessive disorder, visual or auditory hallucinations. Additionally, they show a complex auxo-action between each other or between physical symptoms and mental symptoms.

## Etiology

The factors responsible for the high prevalence of depressive syndromes in ADNS are not fully understood and remain controversial. However, different etiologies of depressive syndromes might point to different treatment strategies, and therefore, affect both treatment success rate and patient outcome. Consequently, this topic deserves increased levels of attention.

### Psychological factors

All chronic disorders, including ADNS, may have psychological consequences during the clinical course of disease. Psychological factors can partly explain the elevated prevalence of depressive comorbidity in all types of chronic diseases, particularly in ADNS. Depression often occurs as a psychological reaction to the limitations of daily life caused by physical illness (56). The high-frequency and long-term disability of ADNS often acts as a traumatic stressor in patients (44). A large number of studies have searched for the possible psychological factors responsible for depression in ADNS, although it has proven difficult to conclude the subjective experience. Irrespective of the limitations, unpredictable course, feelings of helplessness, loss of pleasurable activities, recessive social relationships, significant stress, and inadequate treatment strategies, are all linked to depression to different extents (9, 57–59). The psychological elements may play the most basic role in the development of depression in both ADNS and other chronic diseases; a previous study, involving regression analysis, reported that psychological factors account for 40% of the variance of depression scores (57). Furthermore, psychological factors alone cannot explain the higher incidence of depression in ADNS.

### Depression related to immunological dysregulation

The relationships between immunological dysregulation and psychological function are a progressively important field of study at present for neuropsychiatric diseases. Many research studies into the underlying pathogenesis of major depressive disease have shown that immune activation, and the production of cytokines, may be involved in depression (60, 61). It has also been demonstrated that central or peripheral immune action can trigger depressive behavior by increasing the production of pro-inflammatory cytokines, such as interleukin-1 (IL-1) and IL-6, as well as activating cell-mediated immunity (62). In animal experiments, peripherally-administered pro-inflammatory cytokines IL-1β and TNF-α, as well as lipopolysaccharide (LPS) and synthetic compounds mimicking viral infection [Poly (I:C)], have induced “sickness behavior” is similar to depressive manifestation in human. And that the pro-inflammatory cytokines IL-1, IL-6, and tumor necrosis factor alpha (TNF-α), as well as C-reactive protein (CRP), may contribute to the initiation and progression of psychiatric diseases, such as depression (62–64).

### Depression secondary to structural brain damage

The potential role of structural brain changes in the development of major depressive disorder is receiving increasing levels of attention from scientists; this is particularly driven by the recent advances in medical imaging technology. Several publications have reported that structural lesions in the brain may contribute to the high incidence of depressive syndromes in ADNS. This could be exemplified by a potential link between depression in MS and structural lesions associated with cerebral demyelination, although this association has not been confirmed as yet. Accumulating evidence shows that MS patients with depression tend to present with more severe atrophy of the frontal region and frontal white matter damage, compared to non-depressed MS patients. Goodstein and Ferrell, who reported three cases in which single and multiple depressive episodes preceded neurological symptoms, were the first to hypothesize that MS lesions cause symptoms rather than boost major depression by psychological factors (65). Subsequent studies aimed to specifically investigate this hypothesis. For example, Zorson et al. carried out a 2-years-long follow-up study in 90 MS patients and attempted to correlate their depressive symptoms with quantitative changes of regional and total lesion load, as well as brain parenchymal volumes. These authors found that depressed MS patients showed an obvious reduction in brain parenchymal volumes in the temporal lobes which thus supported the hypothesis put forward by Goodstein and Ferrell (66). This result was also in line with Shen's research which concluded that depressive symptoms were mainly negatively associated with the degree of demyelinating lesions in the limbic system and frontal lobe (67). Shen et al. also noted that gray matter injury could describe clinical depression and disability better than white matter. More recently, Pravata et al. investigated the correlation between cortical and deep gray matter volume and depression. These authors noted that emotional behavior in MS could be convincingly explained by selective circumscribed cortical gray matter degeneration in the orbitofrontal and temporal lobes, which was similar to that seen in patients with major depressive disorder (68, 69). Furthermore, in a very recent paper, van Geest investigated lesion load and gray volume among MS patients with or without depression and held the opinion that depressed MS patients have more severe structural and functional disconnections than non-depressed MS patients (70). Though there is no definitive conclusion as yet, this does provide an underlying mechanism for depression in MS or in ADNS. Additionally, dysfunction at the hippocampal level, which is particularly evident in anti-NMDA receptor encephalitis, may also act on the structures upstream, thus causing emotive dysfunction. Hippocampal dysfunction may affect learning, thinking, memory and therefore frontal executive functions (27). The destruction of glutamatergic receptors and associated proteins in the forebrain and limbic system by anti-NMDA receptor antibodies may also provide direct evidence of an etiological correlation between autoimmunity and the subsequent risk of psychosis, including depression.

### Drug-related depression

Some immunomodulatory drugs, such as interferon (IFN)-beta and steroids, may be blamed for the development of depression. IFN beta, as a form of disease-modifying drug, is commonly used in MS to reduce relapse rates and delay physical disability. Many cases have reported depression after IFN beta treatment (71); this has led to significant research efforts which have attempted to identify whether interferon shows neuropsychiatric toxicity and can induce depression. However, there is no definitive conclusion at present. Patten et al. endorsed the relationship between IFN and depression in his analysis of the incidence of depression in 1,995 patients receiving IFN and 824 patients receiving placebo (72); results showed that the number of patients included in the treatment group reporting depression was twice that of patients taking the placebo. Furthermore, 1.3% of patients developing depression went on to abandon the treatment. This finding was also supported by a range of subsequent studies (61, 73, 74). In contrast, some scientists arrived at a different conclusion and stated that there is no relationship between IFN and depression (75, 76). In fact, some authors have concluded that depression after IFN treatment may be better explained by a previous history of depression (77).

Furthermore, this, steroid may be partly responsible for the depression in MG (78). This hypothesis is exemplified by Suzuki's study in which 287 cases of MG were recruited to investigate the factors underlying depressive states in MG (54). These authors concluded that the dose of oral corticosteroids represented the major factor associated with depressive state in MG, followed by unchanged status, despite treatment and early disease stage. This possibility seems worthy of discussion, as exogenous corticosteroids have been associated with depression among the general population before. Although these links still remain unclear, the existence of a hypothesis relating to a possible relationship has led to the careful management of such patients (79).

The depressive state derived from diverse psychological factors present with more mood change, such as feelings of helplessness, anhedonia and lack of confidence. This status can be improved with the support from others or the improvement and stabilization of neurological symptoms. Organic depressive syndromes, depressive manifestation in ADNS involving brain tissue, show more psychotic feature. They are frequently occur along with other psychiatric symptoms and show poor reaction to the antidepressant. Depression related to immunological dysregulation and medication for ADNS often co-occur with behavior changes. In fact, there are no clear distinction between depressive presentations from different source. More than one element contribute to the development of depression and various components of depressive phenomenology can fluctuate over time.

## Influence

Depression is one of the most important factors that can affect an individual's health. A substantial number of studies has shown that depression increases morbidity, mortality, reduces the quality of life of patients and increases the risk of complications and metabolic problems. Furthermore, the development of a psychiatric condition can increase medical costs, as well as the cost of caring for mental health. This form of adverse effect is strengthened in ADNS. Nowadays, the increased prevalence of depression in ADNS is gradually drawing our attention to the multifaceted burden of depression.

### Reduction of health-related quality of life (HRQL)

A number of research studies have focused upon the reduction of health-related quality of life (HRQL) in patients with ADNS caused by depression. Neurological deficit usually accounts for 40–50% of the reduction in HRQL, while depression, along with fatigue, pain, and cognitive impairment accounts for the remainder. Significant research into the HRQL of MG has documented that psychosocial disorders, predominantly anxiety and depression, are negatively correlated with HRQL, based on multivariate linear regression analysis, apart from significant demographic predicators (older age and lower education) and the current status of myasthenia gravis. Interestingly, the Hamilton Anxiety Rating Scale was verified as a more significant prediction factor for a lower quality of life in both physical and mental aspects than the Hamilton Depression Rating Scale (80–82). Similarly, Shi's research demonstrated that anxiety, disability, fatigue and depression were independent predictors of poor HRQL in NMOSD, and that anxiety was the best predictor of both the global and physical composite scores of HRQL, followed by disability, fatigue and depression (global composite, *r*^2^ = 0.76, *P* = 0.000; physical composite, *r*^2^ = 0.71, *P* = 0.000) (32). This result is in line with previous studies on the depressive condition in NMO (33, 37). Moreover, this type of negative effect is more complex when brain issues are affected. Despite the more severe conditions of ADNS with brain involvement, the burden of a lesion in the brain may modestly correlate to the development of cognitive disability and depression, which may also contribute to a poor quality of life. Taking MS as an example, and excepting the direct impact of the depressive state on the quality of life, depression, as well as fatigue, can cause deterioration in cognitive impairment and exert an indirect impact on the activities of daily living (83). However, the interactions between these factors are intricate. Scientific evidence shows that cognition dysfunctions in many aspects, including verbal memory, sustained attention and concentration and information processing speed, were all associated with depression and fatigue scores to differing extents (84, 85). Nunnari et al. reported that depression score is the most influential variable in terms of higher weight in regression models and the cognitive domains affected (85). Furthermore, symptoms, such as a lack of motivation, an inability to complete tasks, and sleep disturbance, overlap between fatigue, depression and cognitive impairment (86). Thus, it is suggested that recognizing and treating the common comorbidities of fatigue and depression is the first step in diagnosing cognitive dysfunction in a patient with MS (87). Due to the significant effect of anxiety and depression on the quality of life, emotional health should remain a significant clinical focus in patients with ADNS. Such patients should be treated aggressively, especially when cognitive dysfunction exists. Generally, depression is considered to be curable with pharmacological and cognitive-behavioral therapies. If the cognitive impairment persists after the successful treatment of depression, formal neuropsychological evaluation should be carried out.

### Deterioration in physical and mental symptoms

The relationships between physical and other perceived symptoms are also intricate. On the one hand, a depressive condition may contribute to the development and progression of other symptoms and cause further deterioration in these disorders. Depression has also been found to influence cognition in neurological and psychiatric disorders, thus contributing to disability and disease duration (83). Fatigue is the most common physical symptom in ADNS and can be enhanced by a depressive state. This is a very subjective feeling and defined as a reversible, motor and cognitive impairment with reduced motivation and a desire to rest (88). Many studies have found that fatigue is highly correlated with depression and physical impairment and that depressed mood and disability are significant predictors of fatigue in MS, GBS and MG patients (54, 89, 90). The feeling of fatigue will be enhanced in the presence of depression, thus leading to a higher score on the Fatigue Severity Scale (FSS) (17, 91). On the other hand, physical illness can in turn make depression quite probable. Depression symptomatology and prevalence are significantly increased in individuals who have a higher score on the fatigue severity scale (92). This finding is consistent among the general population in that fatigued individuals report a higher proportion of depression symptoms (93).

### Delay of diagnosis

Symptoms of physical disease may partially overlap with depressive symptoms, causing a delay in diagnosis of physical disease or an unnecessary intensive pharmacological treatment. Fatigue and a lack of energy, shortness of breath, and increased weakness of muscles are the prominent symptoms of MG but initially may not be recognized due to their coincidence with depressive symptoms. The comorbidity of psychological symptoms that appears during the course of disease may also be regarded as symptoms of MG. An incorrect understanding of this presentation may render a change of therapeutic strategy which is unnecessary. Psychological manifestation must be carefully treated because of the risk of deterioration in the underlying neurological disease. Furthermore, the incidence of therapeutic drug-related depression in ADNS can also reduce adherence to disease-modifying therapy.

## Conclusion

Because of our limited understanding of the depressive syndromes experienced in patients with ADNS, there is a significant lack of attention and effective treatments at present with which to improve these unpleasant symptoms. In this article, we shed light on depressive syndromes associated with autoimmune disorders of the nervous system and demonstrate the prevalence and clinical profile of depressive symptoms in ADNS. We also discuss the potential mechanisms underlying the high incidence and prevalence of depression in ADNS.

Our study indicates that depression is a common comorbidity in ADNS with a frequency of 15–50% across different types of ADNS; this is higher than the general population and other chronic diseases. Therefore, neurologists should keep this in mind, especially with regards to patients presenting with psychiatric symptoms associated with unexplained neurological findings. The risk factors for depression vary across different disorders but share similar characteristics with major depressive disease. The high frequency of depression in ADNS highlights the need for further research with which to deepen our understanding of the origin of depression. More high-quality literature, with reduced heterogeneity, is now required. This paper uncovered some challenges or key questions for neurologists in diagnosis and treatment of ADNS. First, early discovery to the ADNS symptoms in the context of depressive presentation can be difficult. Second, timely and correct diagnosis as well as proper intervention to the depressive syndrome in ADNS can act as a puzzlement for neurologists. Third, how to address drug-related depression when the related medication is necessary. All these questions need the effort from our peers in this field to develop an integrative strategy in providing appropriate treatment guidelines in future. We hope that this review will promote understanding of depressive syndromes in ADNS, and draw more attention to this clinical problem.

## Author contributions

All authors listed have made a substantial, direct and intellectual contribution to the work, and approved it for publication.

### Conflict of interest statement

The authors declare that the research was conducted in the absence of any commercial or financial relationships that could be construed as a potential conflict of interest. The handling Editor declared a shared affiliation, though no other collaboration, with the authors YL and XT at the time of the review.
